# Impact of Cervical Spine Immobility on Carotid Endarterectomy: A Report of Two Cases

**DOI:** 10.7759/cureus.102202

**Published:** 2026-01-24

**Authors:** Daiki Gohara, Yuta Nakagawa, Yoshihiko Motohara, Akihito Hashiguchi, Koichi Moroki

**Affiliations:** 1 Neurosurgery, Tokuda Neurosurgical Hospital, Kanoya, JPN

**Keywords:** carotid endarterectomy (cea), cervical range of motion (rom), diffuse idiopathic skeletal hyperostosis (dish), internal carotid artery stenosis, ossification of the posterior longitudinal ligament (opll)

## Abstract

Carotid endarterectomy (CEA) is a well-established revascularization procedure for carotid artery stenosis. While anatomical challenges such as high carotid bifurcation and vascular anomalies are widely recognized, cervical spine immobility due to spinal pathology is infrequently considered during preoperative planning. This report presents two cases in which severe cervical immobility, resulting from diffuse idiopathic skeletal hyperostosis (DISH) and ossification of the posterior longitudinal ligament (OPLL), significantly limited distal exposure of the internal carotid artery (ICA) during CEA. The first case involved a 76-year-old man with symptomatic right ICA stenosis and plaque extending to the C3 level. The second case involved a 75-year-old man with asymptomatic severe left ICA stenosis reaching the C2 level. In both cases, imaging demonstrated extensive DISH and OPLL, and cervical immobility impeded distal ICA exposure despite standard techniques. These cases highlight cervical immobility as an underrecognized factor contributing to technical complexity in CEA and emphasize the importance of assessing the range of motion (ROM) limitations during preoperative evaluation for high cervical lesions. Early identification of such limitations may inform perioperative planning and support individualized treatment selection, including consideration of endovascular alternatives when appropriate.

## Introduction

Carotid endarterectomy (CEA) remains a first-line revascularization strategy for most symptomatic carotid stenosis and for appropriately selected asymptomatic lesions when perioperative risk is acceptable, supported by randomized trials and contemporary guidelines [1-4]. In determining operative feasibility, anatomical factors such as high carotid bifurcation, tortuosity, prior cervical radiotherapy, and previous neck surgery are commonly recognized contributors to technical difficulty [4].

Successful completion of CEA requires reliable proximal and distal control of the internal carotid artery (ICA) beyond the cranial extent of the plaque. Standard exposure is achieved through cervical extension and contralateral head rotation [5]. However, functional cervical range of motion (CROM) is not routinely assessed in many preoperative evaluations. Age-related spinal conditions, including cervical spondylosis, diffuse idiopathic skeletal hyperostosis (DISH), and ossification of the posterior longitudinal ligament (OPLL), can significantly restrict cervical extension and rotation [6-8], potentially compromise distal ICA exposure, and increase operative complexity. This report presents two cases in which severe cervical immobility due to DISH and OPLL substantially hindered distal ICA exposure during CEA and discusses implications for perioperative planning and treatment selection.

## Case presentation

At our institution, to avoid potential adverse effects associated with contrast media, preoperative imaging is typically performed by fusing multiple non-contrast datasets: non-contrast cervical computed tomography (CT), carotid three-dimensional time-of-flight magnetic resonance angiography (3D-TOF MRA), and T1-CUBE plaque imaging [9]. Non-contrast cervical CT is acquired using an 80-slice Aquilion scanner (Canon, Tokyo, Japan) with a slice thickness of 0.5 mm. All MR images are obtained using a Signa™ Explore 1.5-T system (GE Healthcare, Milwaukee, WI, USA). The CT and MRI datasets are uploaded to SYNAPSE VINCENT (Fujifilm, Tokyo, Japan), where rigid registration and multiplanar reconstruction are performed. Fusion imaging enables assessment of plaque morphology, the cranial extent of stenosis, and the anatomical relationship between the carotid artery and cervical vertebral levels.

Preoperative evaluation includes assessment of cardiac function and coronary artery status by cardiology, with continuation of antiplatelet therapy (single or dual agents as appropriate) throughout the perioperative period. General anesthesia is administered with oral endotracheal intubation, as nasotracheal intubation is avoided to minimize the risk of nasal or pharyngeal bleeding. The endotracheal tube is secured at the contralateral oral commissure. Motor evoked potential (MEP) monitoring is utilized, and cranial fixation pins are not employed. The operating table is positioned head-down, the neck is extended, the head is rotated approximately 20 degrees contralaterally, and the chin is elevated and secured with tape. A vertical incision is made along the anterior border of the sternocleidomastoid muscle. Using an operating microscope, the common, external, and internal carotid arteries are exposed and dissected. An internal shunt is routinely placed, and after plaque removal, primary closure without a patch is performed. Postoperatively, blood pressure is maintained below 140 mmHg for the first 10 days.

At this institution, in the most recent 100 consecutive CEA cases performed between February 2, 2021, and October 1, 2025, the mean time from skin incision to carotid arteriotomy, which reflects the duration required to achieve secure distal ICA control after patient positioning, was approximately one hour and 48 minutes. This benchmark is descriptive and may vary depending on the surgeon's experience and institutional workflow.

Representative case descriptions​​​​​

Informed consent for publication of this case report was obtained from both patients described below.

Case 1

A 76-year-old man with a body mass index of 25.3, hypertension, and a history of smoking and no prior cervical spine surgery presented to the emergency room with left-sided weakness and hemi-spatial neglect. Brain MRI revealed multiple scattered acute ischemic lesions in the right cerebral hemisphere.

**Figure 1:**
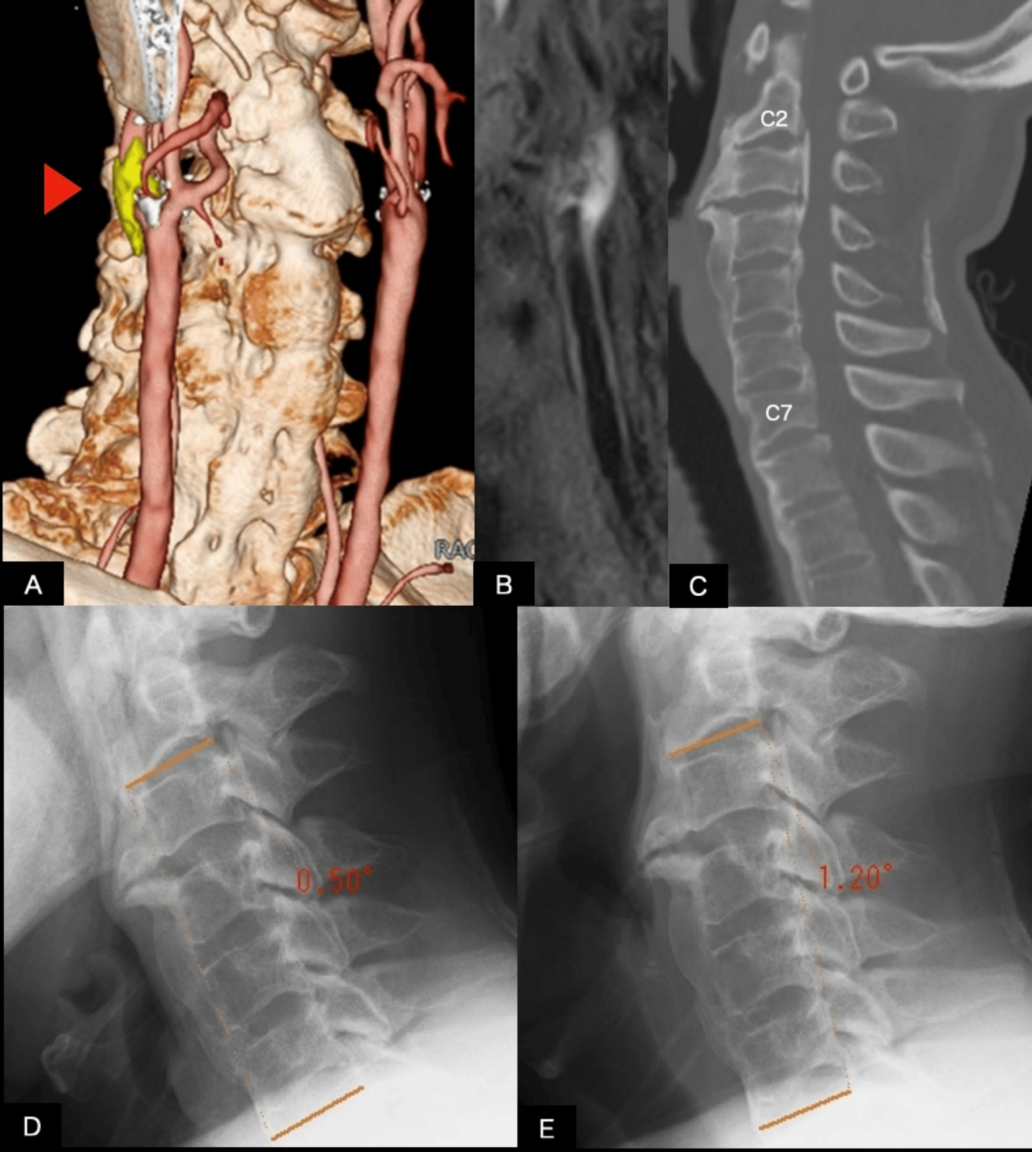
Preoperative multimodal imaging and evaluation of cervical spine immobility in Case 1 (A) Fused non-contrast computed tomography (CT) and magnetic resonance angiography (MRA) image of Case 1. The common carotid artery bifurcation and plaque (arrowhead) are located at the C3 level. Marked anterior bony bridging of the spine is evident; (B) black-blood fat-suppressed T1-weighted MRI demonstrates hyperintensity consistent with intraplaque hemorrhage and increased plaque vulnerability; (C) sagittal CT of the neck shows diffuse idiopathic skeletal hyperostosis (DISH) below the C2 level and ossification of the posterior longitudinal ligament (OPLL) from C2 to C7 levels; (D, E) lateral cervical radiographs obtained in flexion (D) and extension (E). The angle between the inferior endplate of C2 and the superior endplate of C7 measured 0.50° during flexion and 1.20° during extension, resulting in a flexion-extension range of motion of 0.70°, which indicates minimal segmental cervical mobility.

Fusion imaging demonstrated that the distal end of the plaque extended to the C3 vertebral level (Figure 1A). Ultrasonography identified a heterogeneous, echolucent plaque with surface irregularity, while black-blood fat-suppressed T1-weighted MRI showed marked hyperintensity within the carotid plaque (Figure 1B), consistent with intraplaque hemorrhage. These imaging findings are indicative of unstable plaques, characterized by intraplaque hemorrhage, a lipid-rich necrotic core, and increased embolic potential. MRA estimated approximately 70% stenosis. In contrast, duplex ultrasonography demonstrated a peak systolic velocity (PSV) of 88.9 cm/s. The observed lower PSV, potentially attributable to limitations in PSV-based grading and hemodynamic factors, may have led to an underestimation of stenosis severity, as PSV typically exceeds 230 cm/s for stenosis of 70% or greater [10]. Although carotid artery stenting (CAS) is a reasonable alternative for high cervical lesions, CEA was selected in this case because plaque imaging demonstrated unstable plaque characteristics, raising concern for embolic risk associated with CAS.

Cervical CT revealed anterior ossification consistent with DISH extending below the C2 level and OPLL from C2 to C7 levels (Figure 1C). Lateral cervical radiographs obtained in flexion and extension demonstrated markedly restricted cervical mobility, with the angle between the C2 inferior endplate and C7 superior endplate measuring 0.50° during flexion and 1.20° during extension, indicating minimal dynamic change across these segments. Based on these images, the estimated C2-C7 sagittal range of motion (ROM) was 0.70° (Figures 1D, 1E).

**Figure 2:**
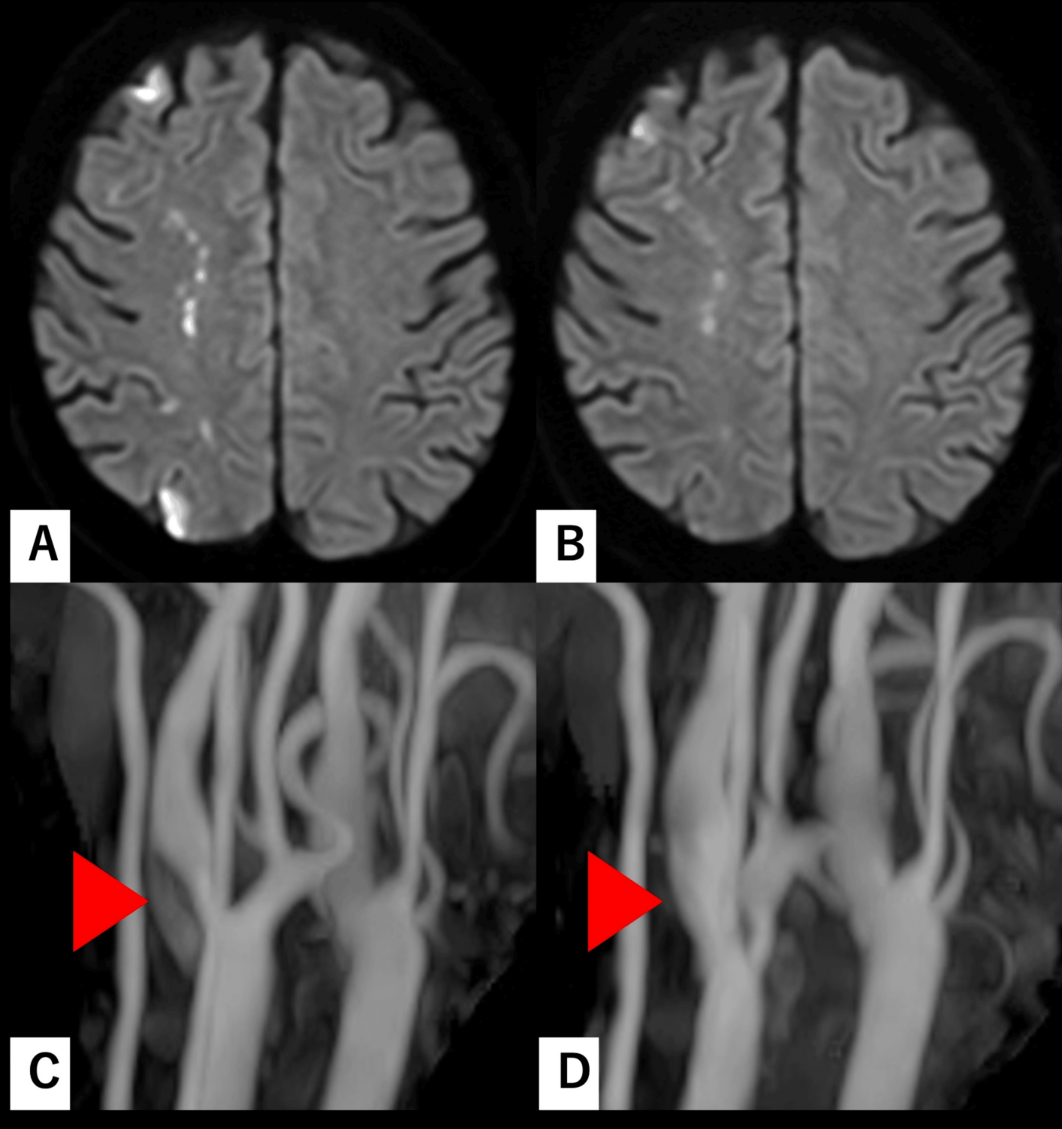
Diffusion-weighted imaging (DWI) and cervical magnetic resonance angiography (MRA) scans of Case 1 before and after CEA Images (A and B) present DWI scans that demonstrate multiple scattered acute ischemic lesions in the watershed zone. Images (C and D) display cervical MRA. Images (A and C) were acquired prior to CEA, on the day of emergency room admission. Images (B and D) were obtained the day after CEA. No additional lesions are observed following the procedure. An increased diameter of the right internal carotid artery (ICA) (arrowhead) and successful vascular reconstruction are evident. CEA: carotid endarterectomy

CEA was performed 19 days after symptom onset. Intraoperatively, cervical rigidity severely limited neck mobility and narrowed the operative corridor. As a result, the time from skin incision to carotid arteriotomy was three hours and 17 minutes, exceeding the institutional mean. Adequate distal ICA control was ultimately achieved without the need for adjunctive high-cervical exposure techniques. Estimated blood loss was 52 mL. Postoperative MRI demonstrated no new ischemic lesions, and follow-up MRA confirmed satisfactory vascular reconstruction (Figure 2). Over approximately six months of follow-up, no recurrent cerebral infarction was observed.

Case 2

**Figure 3:**
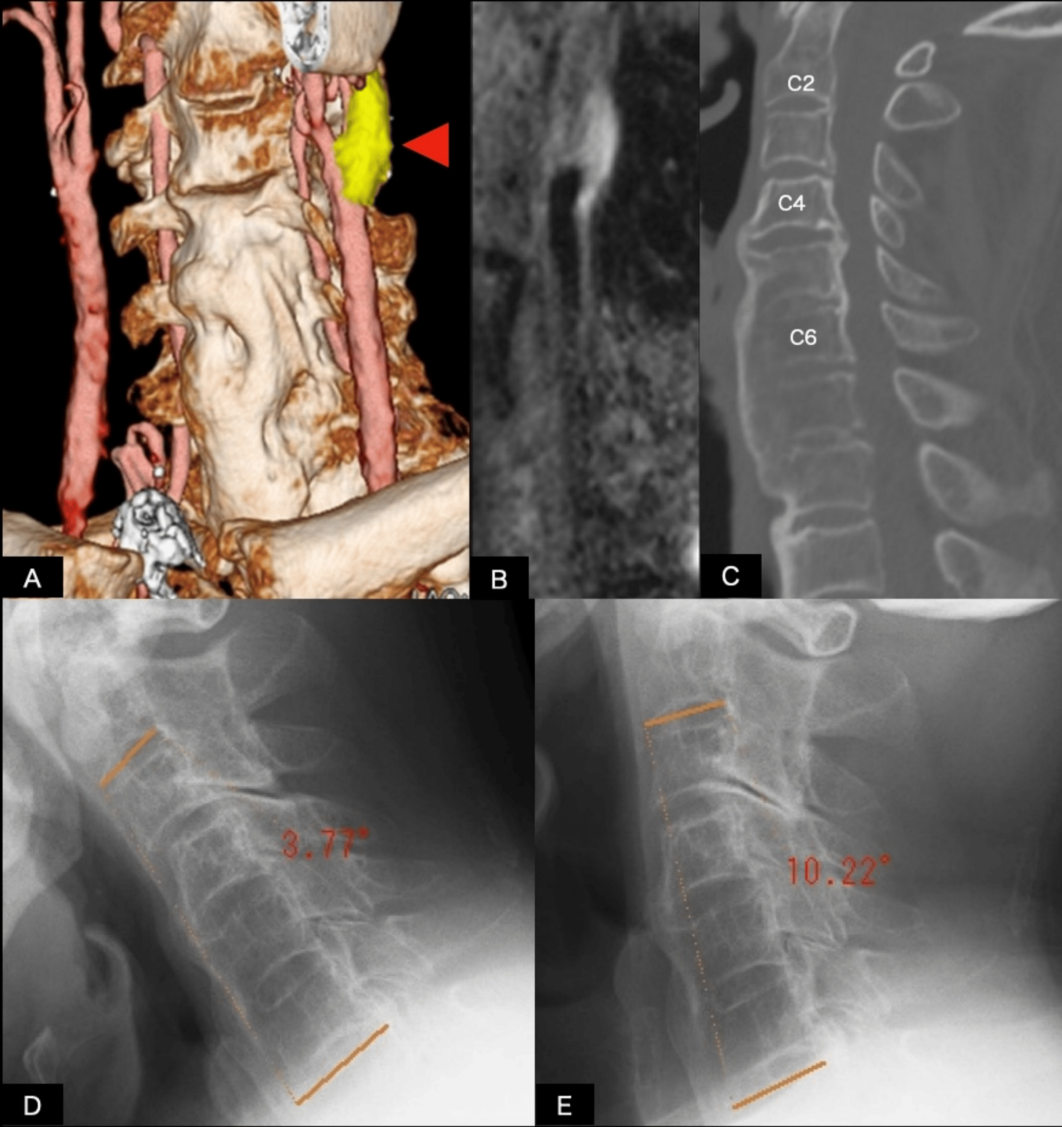
Preoperative multimodal imaging and evaluation of cervical spine immobility in Case 2 (A) Fused non-contrast CT and MRA image of Case 2 shows the common carotid artery bifurcation and plaque (arrowhead). Anterior bony bridging of the spine is also demonstrated; (B) black-blood fat-suppressed T1-weighted MRI demonstrates hyperintensity consistent with intraplaque hemorrhage and increased plaque vulnerability; (C) sagittal CT of the neck shows DISH below the C4 level and OPLL from C2 to C6 levels; (D, E) lateral cervical radiographs obtained in flexion (D) and extension (E). The angle between the inferior endplate of C2 and the superior endplate of C7 measured −3.77° in flexion and 10.22° in extension, crossing 0° between positions. The estimated flexion-extension range of motion was 13.99° (3.77° + 10.22°), demonstrating limited dynamic change. CT: computed tomography; MRA: magnetic resonance angiography; DISH: diffuse idiopathic skeletal hyperostosis; OPLL: ossification of the posterior longitudinal ligament

A 75-year-old man with a body mass index of 30.3, short neck, hypertension, a history of smoking, and prior cerebral aneurysm clipping was monitored through regular follow-up. Surveillance imaging identified bilateral ICA stenosis with large plaques. Fusion imaging demonstrated that the plaque extended cranially to the level of the C2 vertebra (Figure 3A). Black-blood fat-suppressed T1-weighted MRI revealed high signal intensity within the carotid plaque, indicating increased plaque vulnerability (Figure 3B). The left ICA exhibited a PSV of 227.0 cm/s on ultrasonography, and MRI indicated approximately 80% stenosis. In cases involving high cervical lesions, CAS is considered. However, similar to Case 1, the presence of plaque vulnerability led to the selection of CEA over CAS.

Cervical CT identified DISH extending below the C4 level and OPLL extending from C2 to C6 levels (Figure 3C). Lateral cervical radiographs obtained in flexion and extension demonstrated restricted segmental motion, with the angle between the C2 inferior endplate and C7 superior endplate measuring 3.77° during flexion and 10.22° during extension. Because the alignment crossed 0°, between flexion and extension, the estimated C2-C7 sagittal ROM was 13.99° (3.77° + 10.22°) (Figures 3D, 3E).

**Figure 4:**
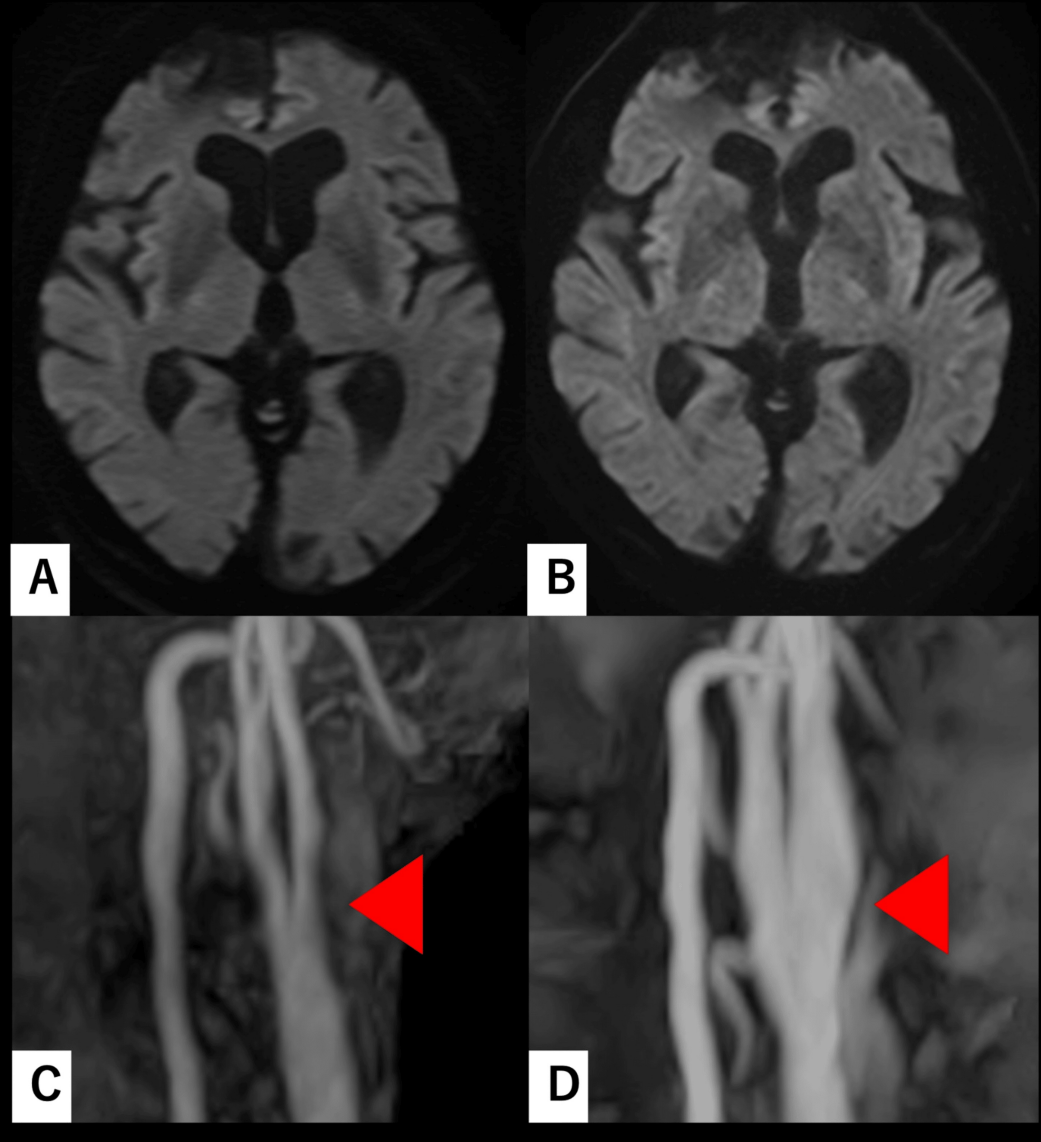
DWIs and cervical MRA images for Case 2 before and after CEA Images (A and B) show DWI scans, while images (C and D) display cervical MRA. Images (A and C) were acquired prior to CEA, and images (B and D) were obtained the day after CEA. No ischemic lesions are observed following the procedure. An increased diameter of the left ICA (arrowhead) and successful vascular reconstruction are evident. DWI: diffusion-weighted imaging; MRA: magnetic resonance angiography; CEA: carotid endarterectomy; ICA: internal carotid artery

Intraoperatively, significant restriction of cervical extension and rotation substantially impeded distal ICA exposure. Extensive cephalad dissection along the sternocleidomastoid muscle and elevation of the parotid tail were required to achieve secure distal ICA control. The time from skin incision to carotid arteriotomy was two hours and 21 minutes, surpassing the institutional mean. Estimated blood loss was 50 mL. Postoperative recovery was uneventful, with no new neurological deficits or lower cranial nerve palsies. Follow-up imaging confirmed successful vascular reconstruction and the absence of new ischemic lesions (Figure 4). Over approximately 18 months of follow-up, no cerebral infarction was observed.

## Discussion

CEA is the first-line revascularization strategy for most symptomatic carotid artery stenoses and for appropriately selected asymptomatic lesions, provided perioperative risk is acceptable [1-4]. In symptomatic ICA stenosis of 70%-99%, CEA reduces the five-year risk of stroke and death by nearly half, and even in 50%-69% stenosis, a significant reduction has been demonstrated [1,2]. Major guidelines consistently recommend CEA over CAS in these settings when perioperative risk is acceptable [4].

These cases illustrate that severe cervical spine immobility due to DISH and OPLL can be a critical and underrecognized factor complicating distal ICA exposure during CEA for high cervical lesions. Safe and reliable completion of CEA requires secure control of the distal ICA beyond the plaque terminus, which is highly dependent on cervical extension and contralateral head rotation. Positioning for CEA is therefore directed at optimizing distal ICA exposure by extending the neck and rotating the head away from the operative side [5]. Cervical extension elevates the mandibular angle and enlarges the operative window between the mandible and clavicle, orienting the distal ICA toward the operative field and facilitating exposure in a more accessible and superficial field [5]. Technical descriptions consistently emphasize this approach as fundamental for achieving adequate distal ICA exposure, particularly in high cervical lesions. Contralateral head rotation complements extension by moving the carotid bifurcation/ICA toward the operative field and improving the working angle for distal manipulation [5]. It also separates the mandible from the ipsilateral shoulder, widening the surgical corridor and expanding the working space for distal dissection. In these cases, multilevel rigidity prevented adequate positioning, resulting in a narrowed operative corridor, limited visualization, and prolonged time to arteriotomy. In Case 2, short-neck morphology and obesity likely compounded these constraints by reducing available working distance and increasing soft-tissue depth.

From a biomechanical perspective, functional cervical extension and rotation are composite motions generated across multiple cervical segments. Sagittal-plane kinematic analyses show that global extension depends on additive motion across subaxial segments, which together increase overall lordosis; restriction in mid- and lower cervical segments can therefore substantially limit effective extension, even if some craniovertebral motion remains [11]. Similarly, in vivo three-dimensional kinematic studies of maximal active head rotation demonstrate that while the upper cervical spine contributes a large proportion of total rotation, additional rotation and coupled motions occur in the subaxial cervical spine. Reduced subaxial mobility therefore constrains global rotation [12]. Multilevel DISH and bridge-type OPLL, both associated with reduced CROM and stiffness [7,8], can markedly reduce achievable operative positioning.

To contextualize the severity of sagittal ROM restriction in these cases, Yukawa et al. reported age-stratified radiographic standards in over 1,200 asymptomatic subjects, showing that mean C2-C7 flexion-extension ROM declines with age from 67.7° ± 17.0° in the third decade to 45.0° ± 12.5° in the eighth decade [13]. In contrast, radiographic C2-C7 ROM in these cases was markedly reduced (0.70° in Case 1 and 13.99° in Case 2), indicating profound limitation relative to age-matched expectations. Although ROM estimates were derived from lateral flexion-extension radiographs rather than standardized multidirectional clinical ROM measurement (such as with a CROM device), and some discrepancy from true functional ROM is possible, the magnitude of reduction supports the clinical impression that the cervical spine functioned as a rigid lever, limiting operative positioning.

Cervical immobility has implications beyond surgical exposure. Limited extension and rotation may complicate airway management and restrict optimal positioning after intubation; difficult airway scenarios have been reported in patients with cervical DISH and extensive anterior osteophytes [14]. In patients with OPLL or advanced spondylosis, forced positioning under general anesthesia may worsen pre-existing canal compromise, increasing the risk of spinal cord compression or ischemic injury. This risk is heightened by muscle relaxation and loss of protective responses under anesthesia, which may allow degrees of positioning not tolerated in the awake state. Therefore, when severe ROM restriction or extensive ossification is identified, perioperative planning may reasonably include cervical spine MRI to assess canal diameter and detect occult cord compression.

Recognition of severe cervical immobility should inform the revascularization strategy. Although guidelines recommend CEA as first-line therapy for many symptomatic patients when perioperative risk is acceptable [4], the feasibility of safe surgical exposure must be weighed against endovascular alternatives. Transfemoral CAS under local anesthesia can avoid forced cervical positioning, but embolic risk and anatomical constraints (such as arch anatomy, calcification, and tortuosity) require careful consideration, particularly when imaging indicates plaque vulnerability. In selected patients and when available, transcarotid artery revascularization (TCAR) may be considered when CEA exposure is expected to be challenging, and CAS is not ideal, as TCAR provides cerebral protection via flow reversal and has demonstrated low perioperative stroke rates in a prospective multicenter trial [15].

Although dedicated reports focusing specifically on cervical immobility in CEA are limited, prior literature has recognized restricted neck mobility as a component of “hostile neck” anatomy that complicates surgical exposure. In a matched case-control series, Frego et al. included a “bull-like and inextensible neck” in the definition of hostile neck anatomy and reported prolonged procedural time in these cases, indicating increased technical complexity during CEA [16]. Similarly, Suliman et al. recorded the presence of a “hostile neck,” defined as prior neck surgery, neck irradiation, or spinal immobility, in a cohort of elderly high-risk patients undergoing CEA [17]. However, previous reports have primarily treated cervical immobility as one element within a composite hostile neck construct, and its specific impact on the operative field, particularly distal ICA exposure, has not been sufficiently articulated. Furthermore, even when ossifying spinal pathology, such as DISH or OPLL, is identified on preoperative CT/CT angiography (CTA), its functional impact, particularly limitations in cervical extension and contralateral rotation, is not routinely quantified and therefore may not be incorporated into preoperative exposure planning. The novelty of the present cases lies not in the detection of DISH or OPLL on CT, but in demonstrating how these often-overlooked findings result in clinically significant exposure difficulty during CEA. Our cases suggest that cervical immobility has the greatest impact in cases involving high cervical lesions that necessitate distal ICA control. Taken together, prior reports and the present cases indicate that severe cervical immobility, although often underemphasized and not routinely quantified during preoperative assessment, can be a clinically meaningful contributor to technical difficulty during CEA, particularly for high cervical lesions.

To address these gaps and translate these observations into clinical practice, incorporating a cervical mobility assessment into preoperative evaluation may benefit patients with high cervical plaques, especially older individuals or those with CT evidence of DISH, OPLL, or severe degenerative changes. Where feasible, objective ROM measurement can standardize assessment; electronic CROM goniometry has demonstrated reliability and validity for measuring cervical mobility [18]. When difficult distal exposure is anticipated, it is essential to recognize that adjunctive exposure techniques for high cervical ICA control have been described, including division or retraction of the posterior belly of the digastric muscle, division of the styloid apparatus, and temporary mandibular subluxation [19,20]. Perioperative planning may also benefit from accounting for the operative environment. A hybrid operating room equipped for both open surgery and endovascular intervention can facilitate procedural transition to an endovascular strategy, such as TCAR or transfemoral CAS, without patient transfer if safe distal control cannot be achieved surgically. This approach requires advanced coordination between open and endovascular teams, availability of appropriate devices and imaging capabilities, and predefined conversion triggers, thereby maintaining procedural flexibility and minimizing delays.

Limitations

This report is limited by the small sample size (two cases), which precludes generalization or statistical inference. Standardized quantitative CROM assessment was not performed; although flexion-extension radiographs provided some evidence of restricted sagittal motion, they do not substitute for validated multidirectional ROM measurement. Furthermore, objective data quantifying the degree of rotational limitation were not obtained, which weakens inferences regarding rotation-specific restriction, despite the association of DISH and OPLL with limitations in both sagittal and axial rotation [7,8]. The assessment of operative difficulty remains subjective and is difficult to quantify in the absence of validated, standardized measures of exposure complexity. Metrics such as exposure time may also be influenced by surgeon experience, intraoperative decision-making, and institutional workflow. The lack of TCAR availability at this institution may further restrict the applicability of these treatment considerations to centers where TCAR is routinely performed. Furthermore, since neither case experienced perioperative neurologic or surgical complications, the impact of cervical immobility on clinical outcomes cannot be determined from these cases. Consequently, the conclusions are limited to operative complexity and preoperative planning rather than procedural risk.

## Conclusions

Based on our experience with these two cases, severe cervical spine immobility resulting from DISH and OPLL can significantly impede distal ICA exposure during CEA for high cervical lesions, thereby increasing procedural difficulty and prolonging procedure time. Because effective extension and rotation are composite cervical motions, DISH and OPLL can critically restrict achievable operative positioning. Targeted preoperative evaluation of cervical pathology and mobility restriction may help anticipate difficult exposure, inform anesthesia and spinal risk assessment, and support individualized treatment selection, including consideration of CAS or TCAR when appropriate.
